# An integrated public health response to an outbreak of Murray Valley encephalitis virus infection during the 2022–2023 mosquito season in Victoria

**DOI:** 10.3389/fpubh.2023.1256149

**Published:** 2023-10-04

**Authors:** Maxwell Braddick, Helen M. O’Brien, Chuan K. Lim, Rebecca Feldman, Cathy Bunter, Peter Neville, Christopher R. Bailie, Grace Butel-Simoes, Min-Ho Jung, Aidan Yuen, Nicole Hughes, N. Deborah Friedman

**Affiliations:** ^1^Communicable Diseases Section, Health Protection Branch, Victorian Department of Health, Melbourne, VIC, Australia; ^2^Victorian Infectious Diseases Service, The Royal Melbourne Hospital, The Peter Doherty Institute for Infection and Immunity, Melbourne, VIC, Australia; ^3^Victorian Infectious Diseases Reference Laboratory, The Royal Melbourne Hospital, The Peter Doherty Institute for Infection and Immunity, Melbourne, VIC, Australia; ^4^Department of Infectious Diseases, Peter Doherty Institute for Infection and Immunity, The University of Melbourne, Melbourne, VIC, Australia; ^5^Agriculture Victoria, Department of Energy, Environment and Climate Action, Melbourne, VIC, Australia

**Keywords:** Murray Valley encephalitis virus, vector-borne disease, mosquito-borne disease, mosquitoes, flavivirus, encephalitis, surveillance, outbreak

## Abstract

**Introduction:**

Murray Valley encephalitis virus (MVEV) is a mosquito-borne flavivirus known to cause infrequent yet substantial human outbreaks around the Murray Valley region of south-eastern Australia, resulting in significant mortality.

**Methods:**

The public health response to MVEV in Victoria in 2022–2023 included a climate informed pre-season risk assessment, and vector surveillance with mosquito trapping and laboratory testing for MVEV. Human cases were investigated to collect enhanced surveillance data, and human clinical samples were subject to serological and molecular testing algorithms to assess for co-circulating flaviviruses. Equine surveillance was carried out via enhanced investigation of cases of encephalitic illness. Integrated mosquito management and active health promotion were implemented throughout the season and in response to surveillance signals.

**Findings:**

Mosquito surveillance included a total of 3,186 individual trapping events between 1 July 2022 and 20 June 2023. MVEV was detected in mosquitoes on 48 occasions. From 2 January 2023 to 23 April 2023, 580 samples (sera and CSF) were tested for flaviviruses. Human surveillance detected 6 confirmed cases of MVEV infection and 2 cases of “flavivirus-unspecified.” From 1 September 2022 to 30 May 2023, 88 horses with clinical signs consistent with flavivirus infection were tested, finding one probable and no confirmed cases of MVE.

**Discussion:**

The expanded, climate-informed vector surveillance system in Victoria detected MVEV in mosquitoes in advance of human cases, acting as an effective early warning system. This informed a one-health oriented public health response including enhanced human, vector and animal surveillance, integrated mosquito management, and health promotion.

## Introduction

**Figure 1:**
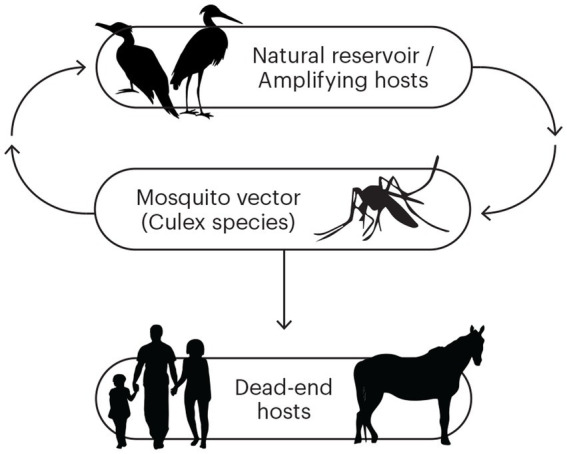
The transmission cycle for Murray Valley encephalitis virus (MVEV). The natural and amplifying hosts are waterbirds, and the primary vector is *Culex* species mosquitoes. Humans and horses are dead-end hosts.

In south-east Australia, the epidemiology of Murray Valley encephalitis virus (MVEV), a mosquito-borne flavivirus, is particularly unusual due to long periods of inactivity punctuated by substantial outbreaks resulting in significant mortality. The virus, which is genetically and antigenically related to Japanese encephalitis virus (JEV), was first isolated from the brain tissue of human cases of encephalitis in 1951 (1). These cases were part of an outbreak of encephalitis centred around the Murray Valley region of south-eastern Australia. The virus has also been implicated in similar outbreaks in 1917, 1919, and 1925 (2), and proven in subsequent outbreaks in 1974 and 2011 (2, 3). Further study identified the freshwater breeding mosquito, *Culex annulirostris*, as the primary disease vector, and water birds, particularly egrets and the Nankeen night heron, as important amplifying animal hosts (Figure 1) (4, 5). Humans, along with terrestrial vertebrates such as horses, are “dead-end” hosts and do not become significantly viraemic to facilitate onward transmission of virus (6).

Human infection with MVEV has an incubation period of 1–4 weeks followed by a variable prodrome of fever and headache (7). The progression to encephalitis is characterised by neurological signs and symptoms which may at first appear non-specific. Only one in every 150–1,000 people infected with MVEV suffer severe disease, with the vast majority of infections being asymptomatic (8, 9). Clinical illness in horses is similar, with a subset of infections progressing to encephalitis and death (10). The illness is difficult to clinically distinguish from infection with JEV and the Australian sublineage of West Nile virus, Kunjin virus (KUNV) and diagnosis relies on serology and molecular methods (7).

Epidemiological and genomic analyses have clarified the ecological patterns of MVEV activity in Australia (11). In the south-east the virus is epizootic, evidenced by infrequent large human outbreaks (12). In the north-west, there are enzootic foci of extant virus circulation evidenced by sporadic human cases and pockets of high seropositivity (9).

The largest outbreak on record in south-east Australia was in 1974, with 58 human cases (27 in Victoria) and a case fatality rate of 20% (13). The most recent epidemic occurred in 2011 with 17 cases across New South Wales (NSW), South Australia (SA), and Western Australia (3). Confirmed human cases were conspicuously absent in Victoria in 2011 despite sentinel chicken seroconversions and a significant equine outbreak (14). In keeping with such infrequent appearances of MVEV in the south-east, there is known to be relatively low seroprevalence amongst people born after 1974 (12).

The dominant explanatory theory of MVEV reintroduction posits that the movement of migratory waterbirds from an area of enzootic activity, under optimal conditions for waterbird and mosquito breeding, allows for amplification of the virus in wildlife and a spill-over phenomena resulting in human cases of infection (15, 16). Consequently, research and public health efforts in the south-eastern states of Australia have focussed on predicting the circumstances that increase the likelihood of outbreaks.

Historically, three climate models, tested against historic outbreaks, have been used to assess the risk for MVEV activity in the Murray Valley region in any given year. Forbes’ hypothesis, based on a historical analysis published in 1978, predicts an outbreak when rainfall averages in four major river basins are above the 7th decile value in the previous summer, and immediate spring/early summer period, preceding an enhanced mosquito season (17). The Nicholls hypothesis uses average mean sea level pressure (MSLP) in Darwin, a surrogate for the Southern Oscillation Index, and the Bennett hypothesis uses a negative Indian Ocean Dipole to predict MVEV activity (18, 19). Given the short historic time frame on which these hypotheses are based, and an imperfect track record of prediction, they are useful yet imprecise elements of a pre-season risk assessment.

Prior to 2021, MVEV and KUNV were the only two encephalitic flaviviruses known to cause locally transmitted disease in Victoria. In March 2021, a case of JEV infection on the Tiwi Islands in northern Australia was identified as a sentinel case (20) preceding an outbreak in the 2021–2022 mosquito season which resulted in 45 cases and seven deaths, centred around the south-east of Australia (21). This outbreak primed the public health system to prepare for further flavivirus outbreaks in the 2022–2023 mosquito season.

Here we describe the 2023 outbreak of MVEV in Victoria, Australia [population 6·7 million (22)] beginning with the pre-season risk assessment and preparedness activities and including the integrated public health response incorporating surveillance activities in humans and animals, laboratory diagnostics, vector control, risk communication and serosurveillance. The Victorian mosquito breeding season typically occurs from October to April each year (23) and collection of surveillance data occurs throughout the season with some programmes beginning before and finishing after the breeding season. Human surveillance data were collected as part of routine notifiable disease reporting and investigation, therefore human research ethics committee approval was not required.

## Pre-season risk assessment

Pre-season mosquito risk assessments directly influence both surveillance and programme activities. The variability in mosquito-borne disease risk is routinely monitored through historic human epidemiology, vertebrate and mosquito surveillance, and predictive climatic factors. The unexpected JEV outbreak in south-east Australia in early 2022 resulted in expansion of mosquito-borne disease surveillance and preparedness for the 2022–2023 season.

The 2022–2023 mosquito season occurred on a background of three sequential years of La Niña weather patterns. By September 2022, water levels in the Murray River had reached the highest level since 1985 (24). October 2022 was the wettest month ever recorded in Victoria resulting in extensive flooding along the Murray, and associated rivers in northern Victoria (25). In addition, rainfall in the Darling, Northern Lake Eyre, and Gulf of Carpentaria basins satisfied Forbes’ hypothesis predicting an outbreak of Murray Valley encephalitis (MVE) in the region of the Murray River (17, 26). The Nicholls hypothesis was also satisfied with MSLP readings in Darwin below the predictive threshold in autumn, winter, and spring of 2022 (18, 26). Further, a negative Indian Ocean Dipole event in 2022 likely contributed to rainfall over south-east Australia and satisfied Bennett’s hypothesis (19). Based on these factors, the relative risk of MVEV activity in Victoria was predicted to be high, compared to previous years.

**Figure 2:**
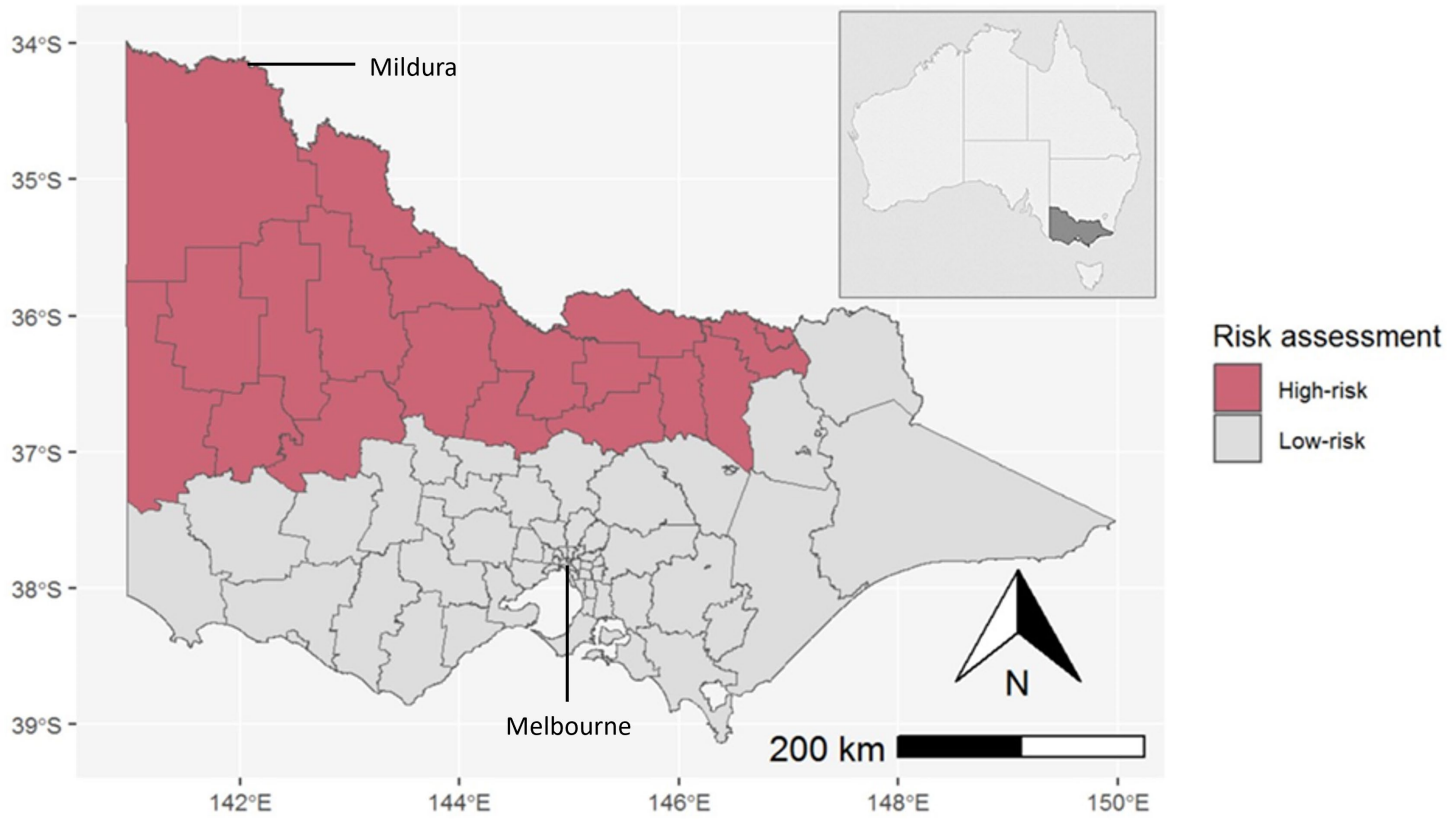
LGAs assessed as high-risk for MVEV/JEV activity in the state of Victoria, Australia (inset). The Murray River runs along the northern border of Victoria.

The geographic scope of predicted risk across Victoria was determined using historic signals of MVEV activity, namely human cases from the 1951 and 1974 outbreaks, equine cases, and sentinel chicken seroconversions. Assuming the potential for an overlapping ecological niche, JEV geospatial modelling (Shearer FM, unpublished) was incorporated into the risk assessment to produce a final composite map of at-risk Local Government Areas (LGAs) (Figure 2).

## Public health response

The public health response to the 2022–2023 MVEV outbreak in Victoria, led by the Victorian Department of Health, was coordinated by an Incident Management Team (IMT) initiated on 28 October 2022.

### Mosquito surveillance

The Victorian Arbovirus Disease Control Program which has operated in Victoria since 1974 funds strategically located local governments in inland and coastal areas considered high-risk for mosquito-borne diseases to undertake regular mosquito surveillance throughout the Victorian mosquito breeding season (27).

The state-wide surveillance system involves monitoring adult mosquito populations on a weekly basis at strategic sites across Victoria, allowing for long-term trend analysis. In inland Victoria, the most common mosquito trap in use is a form of Encephalitis Vector Surveillance trap baited with carbon dioxide and light to attract primarily night-time biting mosquitoes (28). Mosquito specimens are frozen to preserve virus RNA and transported to the Centre for AgriBioscience (Melbourne, Victoria, Australia) for mosquito counting, morphological species identification, and viral testing.

Targeted polymerase chain reaction (PCR) testing is conducted for the presence of three flaviviruses; JEV, MVEV, and KUNV, and two alphaviruses; Ross River virus and Barmah Forest virus (29, 30). In most instances, after mosquito counting and species identification is complete, all mosquitoes in a single trap are re-combined back into their original single, mixed-species pool, and then tested as a “whole trap grind” to accelerate the availability of virus testing results. Where mosquito abundance is high, pool size is limited to 1,000 mosquitoes and multiple pools from a single trap are tested.

Mosquito surveillance results inform updates to local and state-wide risk assessments, identify locations of increased risk to human populations, and inform vector control activities. Programme expansion targeted areas identified as higher risk and without existing surveillance. Further traps were added throughout the season in response to evolving risk, for example into areas impacted by the October 2022 floods in northern Victoria.

Mosquito trapping for surveillance in the 2022–2023 season was expanded from 13 to 22 LGAs (including 315 unique trap sites) across Victoria, leading to a total of 3,186 individual trapping events and a total of 1,027,867 mosquitoes captured between 1 July 2022 and 20 June 2023. Excluding damaged traps and those which collected no mosquitoes, a total of 2,411 traps were analysed and 3,995 PCR tests were performed on whole trap grinds or mosquito species-specific pools (29).

Data demonstrated high to very high levels of mosquitoes from late October 2022 to early January 2023, particularly in LGAs in northern areas of Victoria adjacent or inland to the Murray River. Mosquito numbers declined to moderate and low levels from February 2023.

In high-risk LGAs, a trend was observed with *Culex australicus* [primarily a bird biting species (31, 32)] breeding in very high numbers immediately after flooding, followed by a transition to *Culex annulirostris* [a bird and human biting species (33)] in early December 2022. This provided a pre-warning for the potential of amplification of mosquito-borne viruses within bird populations that can spill over into humans.

**Figure 3:**
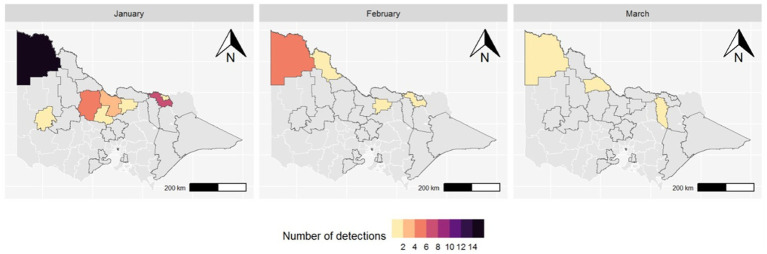
Map of Victorian LGAs with MVEV detected in mosquitoes by month (2023); LGAs outlined in black are those where mosquito surveillance occurred during the season.

MVEV was first detected in a trap in Mildura, the most north-west LGA in Victoria, on 4 January 2023. There were a total of 48 MVEV detections from traps across 11 LGAs between 4 January and 28 March 2023 (Table 1). The highest number of detections consistently occurred in the north-west of the state (Figure 3).

**Table 1:** MVEV detections in mosquito traps by LGA for the 2022–2023 season.

Local government area	Collection date of first detection	Collection date of last detection	Total number of detections
Mildura	4 January 2023	7 March 2023	23
Greater Bendigo	5 January 2023	–	1
Indigo	10 January 2023	15 February 2023	8
Loddon	10 January 2023	31 January 2023	5
Campaspe	17 January 2023	31 January 2023	3
Greater Shepparton	17 January 2023	7 February 2023	3
Horsham	24 January 2023	–	1
Wodonga	24 January 2023	–	1
Swan Hill	7 February 2023	–	1
Wangaratta	15 March 2023	–	1
Gannawarra	28 March 2023	–	1
Total detections (all LGAs)			48

Mosquito surveillance was flexible and adaptive to the situation, whereby additional traps in areas of high risk or areas of confirmed human cases (including potential exposure sites) were quickly deployed to obtain additional information.

Mosquito surveillance and virus screening can provide multiple pieces of intelligence. In addition to presence/absence of virus, testing of multiple pools in a large trap can provide semi-quantitative data about the infection rate of mosquitoes, and testing in species-specific pools can enhance our understanding of vector transmission. However, the latter requires morphological identification of entire traps, and post-flood conditions that lead to significantly increased mosquito numbers impact lab capacity and trap sub-sampling is implemented. This prevents species-specific testing in peak periods. Where resources allowed, some species-specific testing did occur and MVEV was detected in 11 of 19 *Culex annulirostris* specific pools and 1 of 4 *Culex australicus* pools. Species specific testing of *Aedes notoscriptus* (2 pools); *Culex quinquefasciatus* (3 pools); *Anopheles annulipes* (4 pools); *Aedes vittiger* (1 pool); *Aedes theobaldi* (1 pool), and *Culex molestus* (2 pools) all yielded negative results for MVEV.

### Laboratory diagnostics

**Figure 4:**
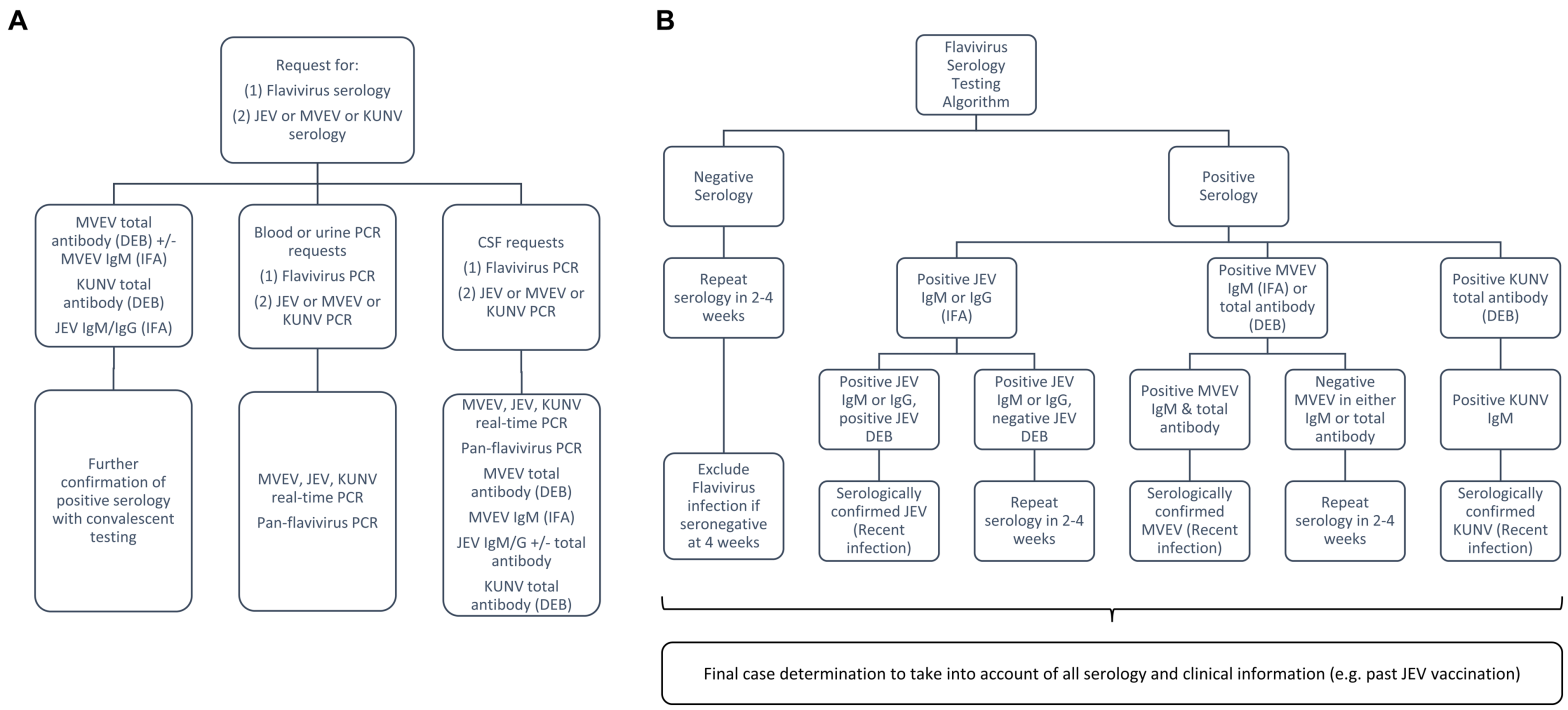
Laboratory testing algorithm for the flavivirus outbreak: **(A)** broad testing approach to capture symptomatic individuals from high-risk areas; **(B)** approach to serological testing and interpretation for those with encephalitis, taking into consideration local epidemiology and vaccination history. MVEV, Murray Valley encephalitis virus; JEV, Japanese encephalitis virus; KUNV, Kunjin virus; DEB, Defined Epitope Blocking Enzyme Linked Immunosorbent Assay (ELISA); IFA, Immunofluorescence Assay.

An enhanced diagnostic approach was implemented as part of the public health response. Due to lack of systematic assessment of serological and viral dynamics from historical outbreaks, the testing algorithm (Figure 4) was designed to both improve case-finding in high-risk areas to characterise the current outbreak and inform future diagnostics. All requests from medical practitioners for flavivirus serology were reflexively followed-up by serological testing for JEV, MVEV, and KUNV, in addition to proactively recommending PCR [blood, urine and cerebrospinal fluid (CSF)] and serology testing (CSF) for encephalitic cases. All flavivirus testing in Victoria took place at the Victorian Infectious Diseases Reference Laboratory (VIDRL) (Melbourne, Victoria, Australia).

MVEV can be detected by molecular techniques on whole blood, urine, CSF and brain tissue, however viraemia is typically brief and serology is often required for diagnosis (7). Enhanced testing on encephalitic cases with strong epidemiological evidence included performing MVEV IgM [Immunofluorescence Assay (IFA)] and MVEV total antibody [Defined Epitope Blocking (DEB) Enzyme Linked Immunosorbent Assay (ELISA)] in parallel and convalescent serology (at least 2–4 weeks apart). DEB is a blocking ELISA using MVEV specific monoclonal antibodies and has been used in Australia with comparable test characteristics to more labour-intensive neutralisation assays (34, 35). Recent MVEV infection was determined by either positivity of both IFA (IgM) and DEB or MVEV antibody seroconversion. Benchmarking of diagnostic assays (molecular, serology, viral culture, and genomics) was performed across multiple public health reference laboratories in Australia as part of the National JEV Diagnostic Project to further inform assay optimisation. Rapid sequencing/genotyping was performed at VIDRL using both Sanger sequencing and next-generation sequencing (Oxford Nanopore) to allow geographical linkage of human and mosquito detections (36).

From 2 January 2023 to 23 April 2023, 580 samples (sera and CSF) were tested for flavivirus antibodies (MVEV DEB, MVEV IFA, KUNV DEB, and JEV IFA; >2,300 tests performed) and flavivirus PCR (Pan-Flavivirus PCR and real-time PCR for JEV, MVEV, KUNV, WNV, yellow fever virus, Zika virus, dengue virus; >2,400 tests performed).

### Human surveillance

MVE is an “urgent” notifiable condition in Victoria under the Public Health and Wellbeing Regulations 2019 and must be notified immediately upon initial diagnosis (clinically suspected or laboratory confirmed) by medical practitioners and laboratories to the Department of Health (37).

The Department of Health urgently follows up all notifications of suspected or confirmed cases of MVE in accordance with national guidelines (38). Interviews with cases under investigation, next of kin and/or treating clinicians are conducted to establish the clinical presentation, potential mosquito exposures, and JEV vaccination status. Treating clinicians are provided with advice on testing, collection, and transport of samples.

Cases are assessed against the Communicable Diseases Network Australia (CDNA) case definitions for MVE and related flaviviruses, where a confirmed case of MVE requires clinical and laboratory (molecular or serological) evidence (39). A risk assessment is undertaken, considering exposure during acquisition period (5–28 days prior to symptom onset), local epidemiology and mosquito surveillance information.

**Figure 5:**
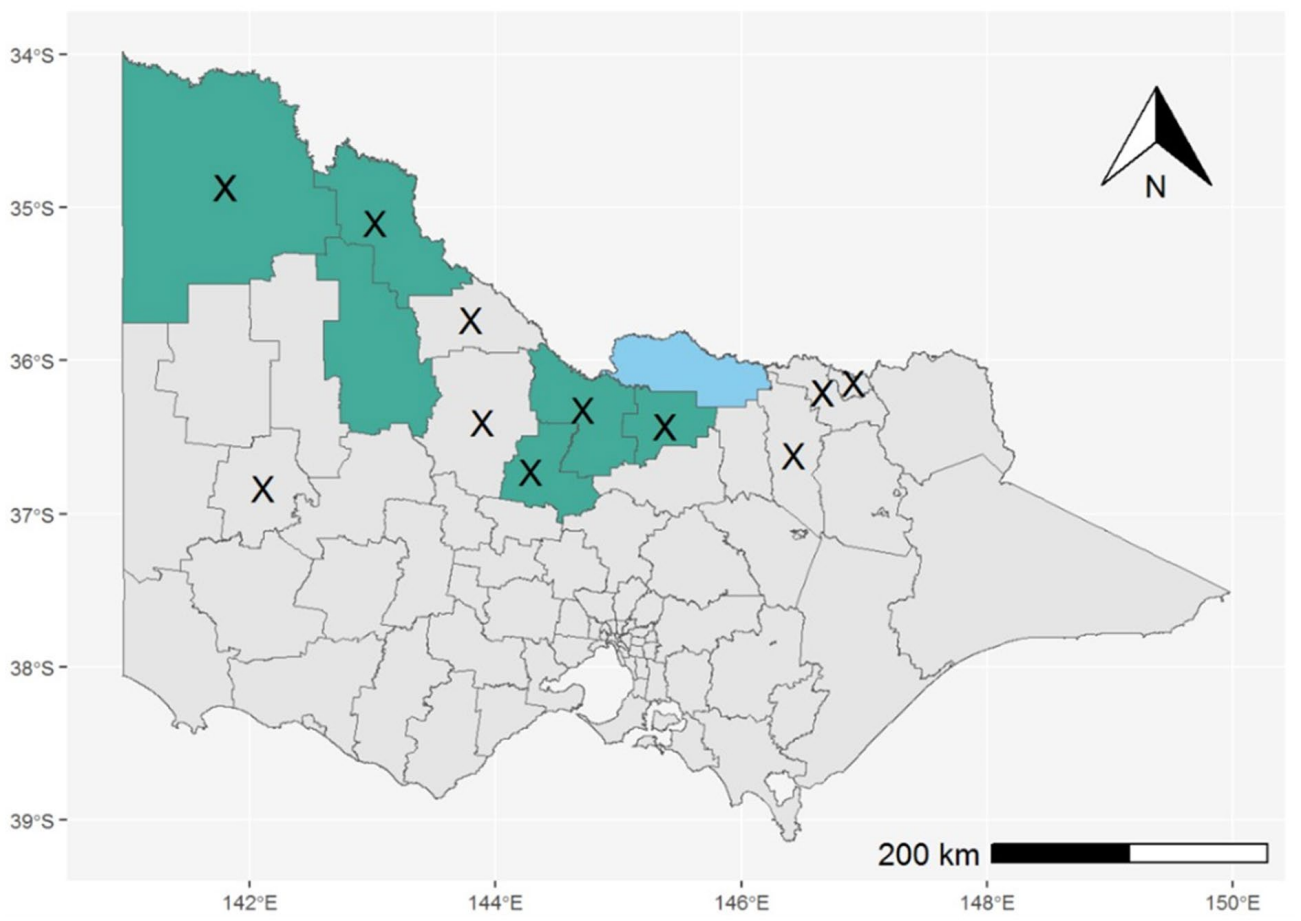
LGAs reported as primary exposure sites for confirmed cases of MVE (shaded green) or “flavivirus infection – unspecified” (shaded blue); LGAs with MVEV detections in mosquitoes are marked with an X.

Throughout the 2022–2023 season (1 October 2022–30 April 2023), the Department of Health received notification of 491 Victorian residents who underwent testing for MVEV. There were six confirmed human MVE cases, of which five were fatal. Two additional recovered cases, with clinical and epidemiological findings consistent with MVE, were classified as “flavivirus infection - unspecified” because of cross-reactivity between MVEV and other flavivirus assays, excluding the ability to differentiate the causative virus, or the presence of co-infection. The median age of cases was 67 years (range 34–72 years) and two of eight cases were female. The range of symptom onset was from 16 January to 29 March 2023. Seven cases were reported to have encephalitis, whilst only one case had a non-encephalitic illness. Possible mosquito bite exposure during the acquisition period for these cases occurred across seven Victorian LGAs, all of which were designated high-risk by the pre-season risk assessment (Figure 5). There were five cases who had also spent time in MVEV risk areas in other Australian states during their acquisition period. Six cases were residents of a high-risk area. Travel to a risk area, fishing, camping, and river swimming were other reported risk factors for exposure. Only one case had a history of prior vaccination against JEV.

Five of eight cases (including both “flavivirus infection – unspecified” cases) were confirmed by serology. The remaining three cases had molecular diagnosis by PCR, one from CSF and two from post-mortem brain tissue, all of which were determined to belong to MVEV genotype 1a, consistent with samples collected from mosquitoes in Victoria.

### Equine surveillance

Given the historic co-occurrence of human and equine MVE outbreaks, Agriculture Victoria collaborated on the state outbreak response.

MVEV is not a notifiable disease in animals in Victoria, however the Victorian Significant Disease Investigation (SDI) Program encourages flavivirus testing by private veterinarians for horses displaying significant or unusual symptoms (40). Initial testing with pan-flavivirus ELISA is performed at the Centre for AgriBioscience, whilst confirmatory testing via serum plaque reduction neutralisation test (PRNT) assay is performed at the Australian Centre for Disease Preparedness (Geelong, Victoria, Australia).

A confirmed case of MVE in a horse is defined as a clinical presentation consistent with flavivirus infection and laboratory results which reveal detection of MVEV antigen via culture, molecular or immunohistochemical methods. In contrast to human case definitions, equine case definitions included a “probable” category which is satisfied by serologic criteria. For probable or confirmed equine cases additional information is collected from the notifying private veterinarian and the owner, including clinical, exposure and travel history. Owners are provided with information and education on prevention and vector control for horses and humans, and encouraged to contact their local public health unit or doctor if they have any health concerns.

From 1 September 2022 to 30 May 2023, 88 horses with clinical signs consistent with flavivirus infection were tested. One probable and no confirmed cases of MVE were detected. The distribution of these horses was state-wide, with tested horses residing across 35 LGAs. At 28 June 2023, three suspected equine cases remained under investigation (awaiting PRNT results to differentiate the causal flavivirus).

### Risk communication

Risk communication and dissemination of key public health messages coordinated by the Department of Health was a core component of the MVE public health response. Risk communication to members of the public impacted by flooding occurred from October 2022 as mosquito numbers boomed across the north-west of the state. A new state-wide health promotion campaign titled “Do not wing it with mosquitoes” was launched in December 2022 featuring new imagery and messaging about mosquito bite prevention (41). A suite of digital, print and other media resources were deployed and translated into 23 languages to cater for culturally and linguistically diverse communities. This included a stakeholder pack containing social media resources for use by local governments and other stakeholders to promote mosquito avoidance. A mosquito-borne diseases webpage was developed as a central repository for all Victorian public information on mosquito-borne diseases, including weekly mosquito surveillance reports and human case surveillance data (42).

Three alerts/advisories were released in late 2022 highlighting the increased risk of mosquito-borne diseases due to flooding and at the beginning of the mosquito season. Further to this, seven reactive alerts and advisories targeting the Victorian community, tourists and health professionals were released in response to new cases and significant surveillance signals throughout the season (43). These provided information on risk, recommendations to reduce risk and detailed clinical and testing information for clinicians.

Targeted risk communication was undertaken through key stakeholders for select populations at higher risk of exposure to mosquitoes such as those with insecure housing, those residing in dwellings without insect screens, populations residing in flood-affected areas, populations displaced by flooding, and children attending early childhood education centres and schools. Clinicians and laboratories were further engaged through webinars to promote clinical awareness of MVE, indications for testing and notification requirements. Human health public awareness campaigns were disseminated to equine and agriculture industry bodies. Targeted communications were also undertaken by local public health units in affected areas.

### Integrated vector control

Integrated Mosquito Management (44, 45) is utilised in Victoria each mosquito season to manage the human health risks associated with mosquito-borne diseases. It is informed by mosquito surveillance data (species and abundance), virus detection in mosquito populations, human case exposure information, environmental conditions, and an assessment of the risk of disease to localised populations. A range of control methods (physical and chemical) are utilised to manage larval and adult mosquito populations, targeted to where and when people are at greatest risk. Integrated mosquito management aims to limit the transmission of pathogens by reducing or eliminating vectors (in this case mosquitoes) from human contact (46).

In Victoria, landowners or land occupiers are responsible for mosquito management on their properties (37), and local governments as large landowners in regional areas play a significant role in local vector control. In the 2022–2023 season, the Department of Health worked collaboratively with local government to support, coordinate, and implement vector control activities in areas of heightened public health risk. This included investment in high-capacity vector control equipment, delivery of vector control training (e.g., in the safe handling and use of chemicals to ensure efficacy of treatments whilst minimising non-target impacts), coordination of resources and expertise across LGAs, and provision of expert advice to enable councils to effectively reduce the risk posed by mosquitoes in their local communities.

Chemical applications including larvicide and adulticide are used by local government to reduce vector numbers where they pose a risk to large human populations. Larviciding, including the use of S-Methoprene and *Bacillus thuringiensis israelensis* (*Bti*) were deployed in habitats where large numbers of larvae were observed. Fogging, involving the application of an adulticide using a natural or synthetic pyrethroid chemical in the form of a fine mist or aerosol, is utilised to target adult mosquitoes, particularly when abundance is elevated, or virus is detected in mosquitoes. Residual barrier treatments through the application of a synthetic pyrethroid to surfaces where adult mosquitoes may land were deployed in areas where people were found to congregate (outdoor school settings, campgrounds, toilet blocks, barbeque, or playground areas). These strategies were implemented proactively based on detailed local knowledge of mosquito breeding sites in proximity to human populations. Intelligence from field observations, mosquito trapping data, and human and animal surveillance data allowed the Department of Health to direct and redeploy appropriate vector control capacity in a pragmatic manner, associated with local risk throughout the season.

## Discussion

The mosquito breeding season of 2022–2023 resulted in the first outbreak of human cases of MVE in the state of Victoria since 1974 and the first outbreak in south-eastern Australia since 2011. Crucially, the outbreak of human cases was preceded by early warning signals from mosquito surveillance, with the virus being detected in mosquitoes from 4 January 2023, 12 days before the first case’s symptoms began, 8 days before sentinel chickens in the adjacent state of NSW first seroconverted (47) and 15 days before sentinel chickens in the adjacent state of SA first seroconverted (48). This demonstrates that vector surveillance, with virus detection, has the capacity to serve as a critical early warning system (49). In Victoria, routine vector surveillance expanded significantly during the 2022–2023 season, plausibly increasing the sensitivity of the system as an early warning tool. The representativeness of trapped mosquitoes is influenced by several factors. These include weather, sample size, and the condition of mosquitoes at the time of collection. Consequently, the methodology of mosquito surveillance must be adaptable throughout the season, such as when flooding renders trap sites inaccessible. Whilst these pragmatic adjustments may confound an isolated evaluation of the system, they represent important learnings in management of mosquito-borne diseases through surveillance in real-world settings.

Vector control activities, performed both pre-emptively and in response to surveillance signals, form an important part of the public health response. Adulticide fogging is the only means of killing adult mosquitoes that are known to be carrying disease. Fogging creates a protective buffer between mosquito populations and residential areas when the disease risk and/or vector abundance is high, and where the use of larvicides is not feasible due to the increased size and extent of breeding sites after major flooding events. However, fogging only temporarily reduces the number of adult mosquitoes. In contrast, barrier treatments applied to surfaces bind, and provide mosquito control for up to 6–8 weeks (50, 51), but cannot be applied on a wide scale and are reserved for smaller, targeted areas.

The real-world nature of the integrated mosquito management programme in Victoria, whilst based on available evidence and best practice principles, is one of a suite of interventions, and therefore direct measurement of the success of vector control in isolation is limited. There is, however, a supportive body of observational evidence showing fogging has been demonstrated to kill 90% of adult dengue mosquitoes (52), and residual surface sprays have been demonstrated to reduce adult mosquito populations by 87–100% for 9 weeks post spraying (53). Further, larviciding has been shown to reduce the next cohort of emerging mosquitoes by approximately 95% (54). Given the high numbers of viral detections in adult mosquito surveillance samples, these evidence-based measures to reduce mosquito numbers present a logical intervention to prevent human disease where human populations are in close contact with mosquito populations.

The MVEV outbreak in 2022–2023 led to high mortality rates amongst confirmed cases of MVE, yielding a case fatality rate of 83%. High mortality is consistent with previous outbreaks and this case fatality rate is higher than in 1974, when 20% of cases succumbed to infection (13). A detailed clinical case series may highlight contributory factors behind case fatality however it is notable that median age for the outbreak was 67 years, compared to a median of 42 years in a case series from 1974 (13). The small number of cases and advances in diagnostics and case definitions further limits comparison between these two outbreaks, yet the findings underscore the population’s ongoing susceptibility to severe disease, supported by a 2011 serosurvey showing a seroprevalence of only 2.2% in high-risk regions (12). The notified clinical cases likely underrepresent the actual burden of infection and repeat serosurveys will be crucial in determining the population’s ongoing vulnerability.

Half a century on from the first isolation of MVEV, no directed therapy nor vaccine is available. The role of JEV vaccination in MVEV prevention, whilst particularly relevant for Australia after the 2021–2022 JEV outbreak (55), is uncertain, with insights from animal studies being mixed (56). Only one clinical case in this outbreak was vaccinated against JEV but had premorbid immune suppression making the significance of vaccination in this case unclear.

Serological interpretation to confirm MVEV infection is fundamentally complex due to cross-reactivity between viruses, persisting antibody response to prior infection or vaccine, and confounding anamnestic responses in the setting of acute infection. On a local level, regular public health and laboratory case conferences guided serological interpretation. Additional enhanced testing was also performed on encephalitic cases, in which MVEV IgM (IFA) and MVEV total antibody (DEB) were performed in parallel, alongside assays for the concurrently circulating flaviviruses JEV and KUNV. Despite post-mortem molecular diagnosis of two MVE cases being clinically complex and delayed, these samples allowed for sequencing, providing valuable genomic information that may improve our understanding of MVEV in Australia.

The propensity for flaviviruses to cause disease in agriculturally significant animals makes surveillance and control a clear one-health priority (14). The signs of MVE in horses can be clinically indistinguishable from other notifiable diseases, most notably Hendra virus. Consequently, on notification of a horse with encephalitis, testing for MVEV, in addition to measures related to suspected Hendra virus, is recommended. When carefully overseen, as described in Victoria, this system may act as a *de-facto* surveillance herd of horses across broad geographic areas evidenced by the testing of 88 horses across 35 LGAs. It is surprising that only one equine case of MVEV infection was diagnosed over the 2022–2023 mosquito season. This may speak to the limitations of testing in horses which, in addition to serological cross-reactivity similar to that in humans, is also limited by sample collection for molecular testing due to risks associated with lumbar puncture and autopsy. Evidence on the difference in host tropism of MVEV in different vertebrates is unavailable but may be another possible explanation for this apparent discrepancy. Horses, like humans are considered dead-end hosts for MVEV and do not contribute to onward amplification of the virus (6). Wild birds, however, are the natural reservoirs for MVEV and whilst not tested during this outbreak, a future serological survey of birds, as has been performed for other flaviviruses, may shed further light on the local epidemiology (5, 57).

Shifts in the distribution of vector-borne diseases due to climate change are the subject of global research and speculation. Direct attribution of any one outbreak to the effects of climate change is difficult, however ecological modelling can highlight areas of future risk (58). There are international examples of significant public health risk from emergent mosquito-borne disease, most famously the spread of West Nile virus throughout North America with ongoing seasonal outbreaks (59). For public health authorities concerned about emerging vector-borne diseases, our climate-informed surveillance programme paired with integrated vector control, targeted health promotion and a one-health focus can provide a preparedness framework for these emerging threats.

## Conclusion

Although outbreaks of MVE are rare and infrequent, they are significant public health events when they do occur. Comprehensive vector surveillance, informed by climate predictions, serves as an essential warning tool to trigger a robust public health response. The potential widespread impact of MVEV and similar vector-borne diseases necessitates improvement-focussed analyses of surveillance activities within a one-health framework. As long as pharmacological interventions for MVE remain elusive, prevention and control of outbreaks will remain the mainstay of reducing the burden of these infections.

## Author contributions

MB: Conceptualization, Data curation, Formal analysis, Methodology, Writing – original draft. HO’B: Conceptualization, Data curation, Formal analysis, Methodology, Writing – original draft. CL: Data curation, Formal analysis, Investigation, Methodology, Writing – original draft, Writing – review & editing. RF: Data curation, Formal analysis, Methodology, Writing – review & editing. CB: Data curation, formal Analysis, Writing – original draft. PN: Data curation, formal Analysis, Investigation, Methodology, Writing – review & editing. CRB: Data curation, Formal analysis, Investigation, Writing – original draft. GB-S: Data curation, Formal analysis, Methodology, Writing – review & editing. M-HJ: Writing – original draft. AY: Data curation, Formal analysis, Investigation, Visualisation, Writing – review & editing. NH: Writing – review & editing. NF: Conceptualization, Supervision, Writing – original draft, Writing – review & editing.

## Abbreviations

CDNA: Communicable Disease Network Australia
CSF: Cerebrospinal fluid
DEB: Defined Epitope Blocking ELISA
ELISA: Enzyme-linked immunosorbent assay
IFA: Immunofluorescence assay
IMT: Incident Management Team
JEV: Japanese encephalitis virus
KUNV: Kunjin virus
LGA: Local Government Area
MSLP: Mean sea level pressure
MVE: Murray Valley encephalitis
MVEV: Murray Valley encephalitis virus
NSW: New South Wales
PCR: Polymerase chain reaction
PRNT: Plaque reduction neutralisation test
SA: South Australia
VIDRL: Victorian Infectious Diseases Reference Laboratory

## References

[1] Burnet FM. Murray Valley encephalitis. Am J Public Health Nat Health. (1952) 42:1519–21. doi: 10.2105/AJPH.42.12.1519, PMID: 13007862 PMC1526305

[2] Mackenzie JS Broom AK. Australian X disease, Murray Valley encephalitis and the French connection. Vet Microbiol. (1995) 46:79–90. doi: 10.1016/0378-1135(95)00074-k, PMID: 8545982

[3] Selvey LA Dailey L Lindsay M Armstrong P Tobin S Koehler AP. The changing epidemiology of Murray Valley encephalitis in Australia: the 2011 outbreak and a review of the literature. PLoS Negl Trop Dis. (2014) 8:e2656. doi: 10.1371/journal.pntd.0002656, PMID: 24466360 PMC3900403

[4] Doherty RL Carley J Mackerras MJ Marks EN. Studies of arthropod-borne virus infections in Queensland: iii. Isolation and characterization of virus strains from wild-caught mosquitoes in North Queensland. Australian journal of experimental biology and medical. Science. (1963) 41:17–39. doi: 10.1038/icb.1963.2 14028387

[5] Marshall ID Brown BK Keith K Gard GP Thibos E. Variation in arbovirus infection rates in species of birds sampled in a serological survey during an encephalitis epidemic in the Murray Valley of south-eastern Australia, February 1974. Aust J Exp Biol Med Sci. (1982) 60:471–8. doi: 10.1038/icb.1982.52 6299259

[6] Hollidge BS González-Scarano F Soldan SS. Arboviral Encephalitides: transmission, emergence, and pathogenesis. J Neuroimmune Pharmacol. (2010) 5:428–42. doi: 10.1007/s11481-010-9234-7, PMID: 20652430 PMC3286874

[7] Knox J Cowan RU Doyle JS Ligtermoet MK Archer JS Burrow JN. Murray Valley encephalitis: a review of clinical features. Diagn Treatment Med J Aust. (2012) 196:322–6. doi: 10.5694/mja11.11026, PMID: 22432670

[8] Anderson SG Donnelley M Stevenson WJ Caldwell NJ Eagle M. Murray-Valley encephalitis; surveys of human and animal sera. Med J Aust. (1952) 1:110–4. doi: 10.5694/j.1326-5377.1952.tb74988.x, PMID: 14909905

[9] Broom AK Lindsay MD Plant AJ Wright AE Condon RJ Mackenzie JS. Epizootic activity of Murray Valley encephalitis virus in an aboriginal Community in the Southeast Kimberley Region of Western Australia: results of cross-sectional and longitudinal serologic studies. Am J Trop Med Hyg. (2002) 67:319–23. doi: 10.4269/ajtmh.2002.67.319 12408675

[10] Kay B Pollitt C Fanning I Hall R. The experimental infection of horses with Murray Valley encephalitis and Ross River viruses. Aust Vet J. (1987) 64:52–5. doi: 10.1111/j.1751-0813.1987.tb16129.x, PMID: 3038067

[11] Knope K Whelan P Smith D Nicholson J Moran R Doggett S. Arboviral diseases and malaria in Australia, 2010-11: annual report of the National Arbovirus and malaria advisory committee. Commun Dis Intell Q Rep. (2013) 37:E55–9. PMID: 23692155 10.33321/cdi.2013.37.1

[12] Williams SA Richards JS Faddy HM Leydon J Moran R Nicholson S. Low Seroprevalence of Murray Valley encephalitis and Kunjin viruses in an opportunistic Serosurvey, Victoria 2011. Aust N Z J Public Health. (2013) 37:427–33. doi: 10.1111/1753-6405.12113, PMID: 24090325

[13] Bennett NM. Murray Valley encephalitis, 1974: clinical features. Med J Aust. (1976) 2:446–50. doi: 10.5694/j.1326-5377.1976.tb130324.x, PMID: 994930

[14] Roche SE Wicks R Garner MG East IJ Paskin R Moloney BJ. Descriptive overview of the 2011 epidemic of Arboviral disease in horses in Australia. Aust Vet J. (2013) 91:5–13. Epub 20121219. doi: 10.1111/avj.12018, PMID: 23356366

[15] Mackenzie JS Lindsay MD Coelen RJ Broom AK Hall RA Smith DW. Arboviruses causing human disease in the Australasian zoogeographic region. Arch Virol. (1994) 136:447–67. doi: 10.1007/BF01321074, PMID: 8031248

[16] Smith DW Speers DJ Mackenzie JS. The viruses of Australia and the risk to tourists. Travel Med Infect Dis. (2011) 9:113–25. doi: 10.1016/j.tmaid.2010.05.005, PMID: 21679887

[17] Forbes JA. Murray Valley encephalitis 1974, also, the epidemic variance since 1914 and predisposing rainfall patterns: Australasian medical publishing company. (1978).

[18] Nicholls N. A method for predicting Murray Valley encephalitis in Southeast Australia using the southern oscillation. Aust J Exp Biol Med Sci. (1986) 64:587–94. doi: 10.1038/icb.1986.62, PMID: 3036054

[19] Bennett N Moran R. Peculiarities of Murray Valley encephalitis (Mve) epidemics in South–Eastern Australia: the Indian Ocean dipole (Iod) as a predictor of epidemics. Victorian Infect Dis Bull Dec. (2009) 12:112–5.

[20] Waller C Tiemensma M Currie BJ Williams DT Baird RW Krause VL. Japanese encephalitis in Australia - a sentinel case. N Engl J Med. (2022) 387:661–2. doi: 10.1056/NEJMc2207004, PMID: 36070717

[21] Australian Government Department of Health and Aged Care. Health alert: Japanese encephalitis virus (Jev). (2023). Available at: https://www.health.gov.au/health-alerts/japanese-encephalitis-virus-jev/ (updated 16 June 2023; cited 2023 June 18).

[22] Australian Bureau of Statistics. National, state and territory population. (2023).

[23] Russell RC. Seasonal abundance of mosquitoes in a native Forest of the Murray Valley of Victoria, 1979–1985. Aust J Entomol. (1986) 25:235–40. doi: 10.1111/j.1440-6055.1986.tb01109.x

[24] Murray Darling Basin Authority. River Murray weekly report: For the week ending Wednesday (2022) 7:2022.

[25] Australian Government Bureau of Meteorology. Annual climate summary for Victoria. (2023).

[26] Australian Government Bureau of Meteorology. Long range weather and climate reports. (2022).

[27] Victorian Department of Health Mosquito Management in Victoria. (2023). Available at: https://www.betterhealth.vic.gov.au/health/healthyliving/mosquito-management-victoria (Accessed June 1, 2023).

[28] Rohe DFR. A miniature battery powered Co2-baited trap for mosquito-borne encephalitis surveillance. Bull Soc Vector Ecol. (1979) 4:24–7.

[29] Natsuda Puttharak MG Ronke J Chandel A Brohier N Wang X O’Riley K. Mosquito and arbovirus surveillance report 2022–2023. Melbourne: Agriculture Victoria Research (2023).

[30] Batovska J Mee PT Lynch SE Sawbridge TI Rodoni BC. Sensitivity and specificity of Metatranscriptomics as an arbovirus surveillance tool. Sci Rep. (2019) 9:19398. doi: 10.1038/s41598-019-55741-3, PMID: 31852942 PMC6920425

[31] Kay BH Boyd AM Ryan PA Hall RA. Mosquito feeding patterns and natural infection of vertebrates with Ross River and Barmah Forest viruses in Brisbane, Australia. Am J Trop Med Hyg. (2007) 76:417–23. doi: 10.4269/ajtmh.2007.76.417, PMID: 17360861

[32] Jansen CC Ritchie SA Van den Hurk AF. The role of Australian mosquito species in the transmission of endemic and exotic West Nile virus strains. Int J Environ Res Public Health. (2013) 10:3735–52. doi: 10.3390/ijerph10083735, PMID: 23965926 PMC3774466

[33] Webb C Doggett S Russell R. A Guide to Mosquitoes of. Australia: CSIRO Publishing (2016).

[34] Hawkes RA Roehrig JT Boughton CR Naim HM Orwell R Anderson-Stuart P. Defined epitope blocking with Murray Valley encephalitis virus and monoclonal antibodies: laboratory and field studies. J Med Virol. (1990) 32:31–8. doi: 10.1002/jmv.1890320106, PMID: 1700805

[35] Hall RA Broom AK Hartnett AC Howard MJ Mackenzie JS. Immunodominant epitopes on the Ns1 protein of Mve and kun viruses serve as targets for a blocking Elisa to detect virus-specific antibodies in sentinel animal serum. J Virol Methods. (1995) 51:201–10. doi: 10.1016/0166-0934(94)00105-p 7738140

[36] Russell JS Caly L Kostecki R McGuinness SL Carter G Bulach D. The first isolation and whole genome sequencing of Murray Valley encephalitis virus from cerebrospinal fluid of a patient with encephalitis. Viruses. (2018) 10:319. doi: 10.3390/v10060319, PMID: 29891797 PMC6024754

[37] Public Health and Wellbeing Regulations (2019). Public Health and Wellbeing Regulations 2019 (Vic). Sect. 7.

[38] Communicable Diseases Network Australia. Murray Valley encephalitis – Series of National Guidelines – Guidelines for public health units. (2013).

[39] Communicable Diseases Network Australia. Murray Valley encephalitis: Australian national notifiable diseases case definition. (2010).

[40] Agriculture Victoria. Significant disease investigation (Sdi) program. (2023). Available at: https://agriculture.vic.gov.au/biosecurity/animal-diseases/significant-disease-investigation-sdi-program (Accessed June 1, 2023).

[41] Victorian Department of Health. Better Health Channel: Protect yourself from mosquito-borne disease (2022). Available at: https://www.betterhealth.vic.gov.au/campaigns/protect-yourself-mosquito-borne-disease (Accessed June 1, 2023).

[42] Victorian Department of Health. Mosquito-borne diseases: Vector-borne disease control. (2023). Available at: https://www.health.vic.gov.au/infectious-diseases/mosquito-borne-diseases (Accessed June 1, 2023).

[43] Victorian Department of Health. Health alerts and advisories. (2023). Available at: https://www.health.vic.gov.au/news-and-events/healthalerts (Accessed June 1, 2023).

[44] Rose RI. Pesticides and public health: integrated methods of mosquito management. Emerg Infect Dis. (2001) 7:17–23. doi: 10.3201/eid0701.010103, PMID: 11266290 PMC2631680

[45] Fouet C Kamdem C. Integrated mosquito management: is precision control a luxury or necessity? Trends Parasitol. (2019) 35:85–95. doi: 10.1016/j.pt.2018.10.004, PMID: 30446394 PMC6503858

[46] Wilson AL Courtenay O Kelly-Hope LA Scott TW Takken W Torr SJ. The importance of vector control for the control and elimination of vector-borne diseases. PLoS Negl Trop Dis. (2020) 14:e0007831. doi: 10.1371/journal.pntd.0007831, PMID: 31945061 PMC6964823

[47] New South Wales Health. Nsw arbovirus surveillance and mosquito monitoring 2022–2023: Report number 15. (2023) 28/1/2023. Report No.

[48] South Australia Health. The south Australian arbovirus and mosquito monitoring report: January 2023. (2023).

[49] Braddick M Yuen A Feldman R Friedman ND. Early detection of Murray Valley encephalitis virus activity in Victoria using mosquito surveillance. Med J Aust. (2023) 219:40–1. doi: 10.5694/mja2.51987 37222082

[50] Cilek J. Application of insecticides to vegetation as barriers against host-seeking mosquitoes. J Am Mosq Control Assoc. (2008) 24:172–6. doi: 10.2987/8756-971X(2008)24[172:AOITVA]2.0.CO;2, PMID: 18437835

[51] Hurst TP Ryan PA Kay BH. Efficacy of residual insecticide Biflex Aquamax applied as barrier treatments for managing mosquito populations in suburban residential properties in Southeast Queensland. J Med Entomol. (2014) 49:1021–6. doi: 10.1603/ME11278 23025182

[52] Wang J-H Wu J-W Liu D. Benefit evaluation of dengue adult mosquito chemical control and its application. Taiwan Epidemiol Bull. (2009) 25:391–9.

[53] Muzari OM Adamczyk R Davis J Ritchie S Devine G. Residual effectiveness of Λ-Cyhalothrin Harbourage sprays against foliage-resting mosquitoes in North Queensland. J Med Entomol. (2014) 51:444–9. doi: 10.1603/me13141, PMID: 24724295

[54] De Little SC Williamson GJ Bowman DM Whelan PI Brook BW Bradshaw CJ. Experimental comparison of aerial Larvicides and habitat modification for controlling disease-carrying Aedes Vigilax mosquitoes. Pest Manag Sci. (2012) 68:709–17. doi: 10.1002/ps.2317, PMID: 22076747

[55] McGuinness SL Lau CL Leder K. The evolving Japanese encephalitis situation in Australia and implications for travel medicine. J Travel Med. (2023) 30:taad029. doi: 10.1093/jtm/taad029, PMID: 36869722 PMC10075061

[56] Lobigs M Larena M Alsharifi M Lee E. Pavy M. live chimeric and inactivated Japanese encephalitis virus vaccines differ in their cross-protective values against Murray Valley encephalitis virus. J Virol. (2009) 83:2436–45. doi: 10.1128/jvi.02273-08 19109382 PMC2648276

[57] Coroian M Silaghi C Tews BA Baltag E Marinov M Alexe V. Serological survey of mosquito-borne arboviruses in wild birds from important migratory hotspots in Romania. Pathogens. (2022) 11:1270. doi: 10.3390/pathogens11111270, PMID: 36365021 PMC9699478

[58] Furlong M Adamu A Hickson RI Horwood P Golchin M Hoskins A. Estimating the distribution of Japanese encephalitis vectors in Australia using ecological niche modelling. Trop Med Infect Dis. (2022) 7:393. doi: 10.3390/tropicalmed7120393, PMID: 36548648 PMC9782987

[59] Kretschmer M Ruberto I Townsend J Zabel K Will J Maldonado K. Unprecedented outbreak of West Nile virus - Maricopa County, Arizona, 2021. MMWR Morb Mortal Wkly Rep. (2023) 72:452–7. doi: 10.15585/mmwr.mm7217a1 37104168

